# Impact of Schistosome Infection on *Plasmodium falciparum* Malariometric Indices and Immune Correlates in School Age Children in Burma Valley, Zimbabwe

**DOI:** 10.1371/journal.pntd.0000882

**Published:** 2010-11-09

**Authors:** Davison T. Sangweme, Nicholas Midzi, Sekesai Zinyowera-Mutapuri, Takafira Mduluza, Marie Diener-West, Nirbhay Kumar

**Affiliations:** 1 Department of Molecular Microbiology and Immunology, Johns Hopkins Bloomberg School of Public Health, Baltimore, Maryland, United States of America; 2 Schistosomiasis Section, National Institute of Health Research, Harare, Zimbabwe; 3 Department of Medical Microbiology, College of Health Sciences, University of Zimbabwe, Harare, Zimbabwe; 4 Biochemistry Department, Faculty of Medicine, University of Zimbabwe, Harare, Zimbabwe; 5 Department of Biostatistics, Johns Hopkins Bloomberg School of Public Health, Baltimore, Maryland, United States of America; 6 Department of Tropical Medicine, School of Public Health and Tropical Medicine, Tulane University, New Orleans, Louisiana, United States of America; Queensland Institute of Medical Research, Australia

## Abstract

A group of children aged 6–17 years was recruited and followed up for 12 months to study the impact of schistosome infection on malaria parasite prevalence, density, distribution and anemia. Levels of cytokines, malaria specific antibodies in plasma and parasite growth inhibition capacities were assessed. Baseline results suggested an increased prevalence of malaria parasites in children co-infected with schistosomiasis (31%) compared to children infected with malaria only (25%) (p = 0.064). Moreover, children co-infected with schistosomes and malaria had higher sexual stage geometric mean malaria parasite density (189 gametocytes/µl) than children infected with malaria only (73/µl gametocytes) (p = 0.043). In addition, a larger percentage of co-infected children (57%) had gametocytes as observed by microscopy compared to the malaria only infected children (36%) (p = 0.06). There was no difference between the two groups in terms of the prevalence of anemia, which was approximately 64% in both groups (p = 0.9). Plasma from malaria-infected children exhibited higher malaria antibody activity compared to the controls (p = 0.001) but was not different between malaria and schistosome plus malaria infected groups (p = 0.44) and malaria parasite growth inhibition activity at baseline was higher in the malaria-only infected group of children than in the co-infected group though not reaching statistical significance (p = 0.5). Higher prevalence and higher mean gametocyte density in the peripheral blood may have implications in malaria transmission dynamics during co-infection with helminths.

## Author Summary

Malaria and schistosomiasis are the most prevalent tropical diseases in sub-Saharan Africa and together exert a huge burden of mortality and morbidity. The geographical overlap of these diseases among the individuals and at the population level commonly occurs resulting inevitably in frequent co-infections. It is not clear how helminth infections affect the outcome or the course of malaria caused by *P. falciparum*. Previous studies have suggested both exacerbation as well as suppression of symptoms due to one or both pathogens. Thus the possibility of synergistic or antagonistic interactions needs to be taken into account in planning and implementing interventions, so as to adjust control priorities and strategies accordingly. The most notable impact of schistosome co-infection revealed by our studies was at the prevalence of sexual and asexual stage malaria parasites, which may have implications on malaria disease severity and transmission dynamics.

## Introduction

Malaria and schistosomiasis are the most prevalent tropical diseases in sub-Saharan Africa and together exert a huge burden of mortality and morbidity as well as contributing to underdevelopment of already disadvantaged populations[1]. About 500 million clinical cases of malaria are reported each year whereas at least 200 million people are infected with schistosomiasis[2]. The geographical overlap of these diseases commonly occurs resulting inevitably in frequent co-infections. It is not clear how helminth infections affect the outcome or the course of malaria caused by *P. falciparum*.

Polyparasitism appears to be the rule, rather than the exception, both at the population level and among individuals residing in developing countries [3]. Thus, polyparasitism represents co-endemicity in an epidemiological sense and simultaneous infections (co-infections) in individual patients in a clinical sense. The effects of polyparasitism are often clinically inapparent. However, in some situations, co-infections may exacerbate disease symptoms due to one of the pathogens. Co-existent infections may also, under some circumstances, suppress clinical symptoms due to one or both pathogens. Thus the possibility of synergistic or antagonistic interactions needs to be taken into account in planning and implementing interventions, so as to adjust control priorities and strategies accordingly.

Buck and colleagues reported that helminth infections affect the clinical manifestations of malaria, specifically that subjects infected concurrently with *S. mansoni* and malaria had significantly greater rates of hepatosplenomegaly compared to those affected by either disease singly [4]. However, the general conclusion that helminths exacerbate malaria was challenged by other studies done at the same time [5], [6].

Subsequent investigations revealed a wide range of disparities in findings, fueling further research in the area. Many studies found that helminths increased susceptibility to malaria [5], [7]–[11], whereas others found no such effect [12]–[14], and still others reported lower rates of malaria infection during co-infection [15]–[18]. However, results on schistosome infections indicate that light schistosome infections might be protective in young children as indicated in one study in which Lyke and colleagues reported that children aged 4–8 infected with schistosomes showed less malaria, increased time to first clinical infection and lower parasitemia compared to non-infected controls, although this did not apply to older children [15]. In terms of pathological outcomes, increased hepatosplenomegaly has been reported in intestinal schistosome-malaria co-infected individuals [10], [19], [20], while others reported a protective effect of helminth infection against development of cerebral malaria [16] and acute renal failure [17]. Due to these contrary findings, firm conclusions regarding the nature of helminth-malaria interactions have remained elusive.

There is growing evidence for the protective role of IgG in *P. falciparum* infection. Passive transfer of immunoglobulin G (IgG) has provided protection against *P. falciparum* blood stage in South American (Saimiri) monkeys [21], [22] and in humans [23], [24]. Furthermore, human antibodies efficiently inhibit in vitro *P. falciparum* proliferation [23] and mediate opsonization of infected RBCs [22], a fact exploited in growth inhibition assays. Cytophilic antibodies (IgG1 and IgG3) are currently thought to be protective whereas non-cytophilic antibodies (IgG 2 and IgG4) against the same epitopes are not protective and may instead competitively block the protective activity of cytophilic ones [21], [22], [25]. In areas where malaria is endemic, cytophilic antibodies have been associated with lower parasitemia [26] or lower risk of malaria attack [27]. It is of utmost interest to find out how concomitant schistosome infection affects antibody isotype switching and balance and ultimately susceptibility or resistance to *P. falciparum* malaria.

We have previously described polyparasitism in the study population comprising children in rural areas of Zimbabwe [28]. During the course of these investigations we found that there is an extensive overlap of schistosomiasis and malaria in a number of areas, which include Burma Valley in eastern Zimbabwe as did others [29], Lake Kariba shores [30] and the Southeastern Lowveld Estates near Chiredzi [31]. These areas present an opportunity for the study of the interaction of malaria and schistosomiasis under field conditions in human subjects. In this study the aim was to investigate how schistosome infection affects malaria parasite prevalence, density and distribution in the study subjects. The impact of co-infection on hemoglobin and malaria antibody levels and the impact of schistosome treatment with praziquantel in the children were examined.

## Materials and Methods

### Study design

**Figure 1:**
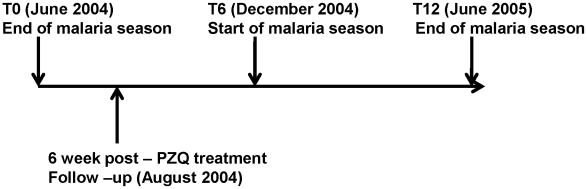
Study Design. The arrows indicate the different times at which urine, blood and stool samples were obtained from study participants for diagnosis. The malaria seasons at baseline and follow up times are indicated as well as treatment of schistosome infected children with praziquantel (PZQ).

The study was a prospective 12-month follow-up of a cohort of children from the end of one malaria season to the end of the next malaria season (Figure 1). At each of the two subsequent 6-month follow up time periods the same parameters were measured and infected children were given appropriate recommended treatment. In total, 605 children aged 6–17 years were recruited at baseline (T0). Each child provided 3 urine samples on 3 consecutive days for *S. haematobium* diagnosis and quantitation, a single stool specimen for *S. mansoni* and other helminth diagnosis, and a further two stool specimens for quantitation. Blood was drawn for malaria and anemia diagnosis. Based on parasitological diagnosis at recruitment, children were grouped as uninfected (N), schistosome-only infected (S), malaria-only infected (M) or schistosome and malaria co-infected (S+M). The same children were subsequently followed up at two further time points of 6 months (T6) and 12 months (T12), respectively for diagnosis of malaria and schistosome infections and collection of blood samples for other study parameters.

### Study area and population

The study area is about 40 km east of the city of Mutare and close to the border with Mozambique. The Burma Valley area is located in the eastern province of Manicaland with a longitude of 32° 48′, latitude of 19° 50′ and altitude of 4, 200 m. The area is mesoendemic for malaria with peak transmission during the rainy season (November to May) with lower level transmission throughout the year. Schistosomiasis is also endemic in the area with transmission mainly in the dry July to October period. This is a commercial farming area in which irrigation practices aid the transmission of both malaria and schistosome parasites. The study participants were drawn from children who attended three elementary schools (Valhalla, Msapa and Kaswa) within a 10-mile radius in proximity to Burma Valley Clinic, which provides health services to the population. These children predominantly belonged to low-income families who were employed as laborers in the estates. Only children aged 6–17 years, attending the three schools and who had been resident in the area for 4 years or more were included in the study.

### Ethical considerations

Samples for this study were obtained from children who agreed to participate following the granting of written informed consent by their parents. Samples were collected with ethical clearance from the Medical Research Council of Zimbabwe and the Johns Hopkins School of Public Health Institutional Review Board (USA) (Study number H.26.04.05.20.B2).

### Parasitological techniques

Urine samples were collected from each participant between 10 am and 2 pm, on three consecutive days to account for any diurnal variations in egg shedding by the urine filtration technique [32]. Briefly, 10 ml of urine were forced through a reusable monofilament polyamide (Nytrel) filter (pore size 12–20 µm) using a syringe. Eggs of *S. haematobium* (size, 150 by 60 µm), if present, are unable to pass through the filter. The filter membranes were stained with Lugol's iodine to enhance the visibility of eggs under the microscope. Children were diagnosed as schistosome infected if *S. haematobium* eggs were detected in any one or more of their 3 urine samples or schistosome free if none were found.

The formal ether concentration method was used to diagnose *S. mansoni* infections and other soil transmitted helminths (STH) (*Necator americanus, Ascaris lumbricoides* and *Trichuris trichiura*) infections from participants' samples as described elsewhere [28]. Children were diagnosed as schistosome infected if *S. mansoni* eggs were detected in their stool samples or schistosome free if not, and children who were STH infected were also treated but excluded.

About 5 ml of venous blood was collected from each participant into two tubes (EDTA coated and plain tubes). Plasma and serum were stored at −20°C for use in further laboratory tests for antibody and cytokine levels and their ability to inhibit malaria parasite growth. Malaria blood smears were prepared from EDTA blood for diagnosis and determination of *P. falciparum* burden. *P. falciparum* parasitemia was estimated by counting the number of malaria parasites in random microscope fields containing 200 white blood cells (WBCs) and then multiplying the result by 40 assuming 8000 WBCs/µl [33]. The detection of any *P. falciparum* malaria blood stage parasite was conclusive of malaria infection. Gametocyte counts were also recorded. Hemoglobin levels were measured for each subject using the Hemocue digital counter (mg/dl). The children were classified as anemic or non-anemic according to the WHO age/gender cut-off limits[2].

### Treatment of infected children

Children who were infected with schistosomes were treated with a single dose of praziquantel (40 mg per kilogram of body weight), whilst those who had STHs received a single 400 mg dose of albendazole (but excluded from further analyses). Children who tested positive for malaria were treated at the Burma Valley Clinic with a combination of chloroquine and sulfadoxine/pyrimethamine in accordance with Ministry of Health and Child Welfare (Zimbabwe) guidelines (2002).

### Total IgG to crude *P. falciparum* antigen by ELISA

*P. falciparum* culture (NF54) was grown *in vitro* and crude antigen (mixture of all parasite blood stages) harvested for use in ELISA detection of antibody levels in plasma from the study participants at the different time points. Cultured parasites were harvested by centrifugation (800 g, 5 min) and released from RBCs by treatment with 0.1% saponin (10 min). Parasites were pelleted (1,800 g, 10 min) and lysed by sonication. The supernatant was collected after centrifugation at 10, 000 g for 20 min and protein concentration measured by the bicinchoninic acid assay (BCA). A 100 µl volume per well of 2 µg/ml of *P. falciparum* crude antigen was used to coat 96-well plates (Immulon 4 HBX, USA) overnight at 4°C. Plate was washed 4 times (PBS/0.05%Tween-20) to remove unbound antigen followed by addition of 200 µl of 5% milk/0.1% Tween to block antigen free surfaces of the wells for 1 hr. After washing the plates 4 times, 100-µl plasma samples (at a dilution of 1:200) were added to each well in duplicate. Plates were incubated at room temperature for 2 hrs on a rocking platform followed by 4 washes. Secondary goat anti-human IgG (Kirkegaard and Perry Labs, MD, USA) conjugated to horseradish peroxidase (HRP) at 1:1000 dilution was added (100 µl/well) and the plates incubated at room temperature for 1 hr. Plates were washed and color developed by adding 100 µl of substrate 2, 2′-azino-di-(3 ethylbenzthiazoline sulfonic acid (ABTS, Kirkegaard and Perry Labs, MD, USA) per well and incubated for 30 min in the dark. Absorbance was read at 405 nm using a multi-scan plate reader and results expressed as optical density (OD) values and recorded as ratios compared to a batch of common pooled sera from malaria exposed adults, which served as the positive control to allow for comparability of samples run on different days and different plates. Pooled normal human serum (NHS) obtained from 12 subjects from the USA with no prior exposure to malaria served as negative control serum. The results were expressed as OD ratios by the formula below:



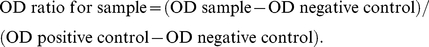



### Total IgG, IgG2 and IgG3 to MSP-1 and AMA-1

To quantify IgG isotypes to the merozoite surface protein-1 (MSP-1) and the apical membrane antigen-1 (AMA-1), the same ELISA protocol as above was used. MSP-1_42_ (FVO) and AMA-1 (FVO) (100 µl per well 1 µg/ml) used to coat plates were provided by Dr S. Singh of the Malaria Vaccine Development Branch, National Institutes of Health and Dr D. Lanar of Walter Reed Army Institute, respectively. For determination of IgG isotypes, the plates were developed with peroxidase conjugated sheep anti-human IgG2 or IgG3 (The Binding Site Limited, Birmingham, UK) at 1 µg/ml.

### *In vitro* growth inhibition assessment by lactate dehydrogenase (LDH) assay

Antibodies that recognize asexual parasite surface antigens such as apical membrane antigen-1 (AMA-1) and merozoite surface protein-1 (MSP-1) have been shown to inhibit parasite growth *in vitro* [34]. Therefore, the functional activity of antibodies (in plasma samples), was characterized by *in vitro* growth inhibition assays (GIAs). *P. falciparum* efficiently utilizes acetyl pyridine adenine dinucleotide (APAD) as a co-factor for the formation of pyruvate from lactate [35] and hence the LDH assay [36], validated to match microscopy [37], was used to determine growth inhibition activity of plasma samples from study subjects. *P. falciparum* parasites (W2 strain which is multi-drug resistant [38], [39] at 0.3% parasitemia) were cultured according to Trager and Jensen's method [40] in RPMI-1640 (GIBCO, USA) medium supplemented with 10% inactivated (30 min at 56°C) normal human serum (NHS), 50 mg/L hypoxanthine (Sigma, USA), 25 mM HEPES (N-2 hydroxyethyl piperazine-N′-2 ethane sulfonic acid) (Calbiochem, USA) and 2.0 mg/ml sodium bicarbonate (J.T Baker, NJ, USA) at 2.5% hematocrit. The use of the drug resistant W2 strain instead of the NF54 strain was to preclude the need for plasma sample dialysis to ensure that any residual anti-malarial drugs would not affect GIA [37]. The parasites were gassed with 5% O_2_, 5%CO_2_ and 90% N_2_ and incubated at 37°C.

Parasites were synchronized once by 5% D-sorbitol treatment [41] prior to GIA. Cultures were pelleted by centrifugation at 1,500 rpm for 5 min, supernatant replaced with twice the pellet volume of 5% sorbitol (w/v in water) and incubated for 10 min at 37°C. The sorbitol was removed by centrifugation and the pellet washed once with medium and parasites cultured in fresh medium. After 40 hr of culture, parasitemia was adjusted to 1% (final concentration) with at least 80% of the parasites at the schizont stage and plated (200 µl total volume) into 96-well Costar flat-bottom well plates (Cambridge, USA) in the presence or absence of test plasma at 1:10 dilution. All plasma samples were tested in duplicate with positive and negative controls included. The plates were incubated in 5% CO_2_, 5% O_2_ and 90% N_2_ using an incubator at 37°C for 48 hr before the lactate dehydrogenase assay was carried out.

At the end of the 48 hr growth period, a fixed volume of parasites (50 µl) was transferred from each well into a microcentrifuge tube and spun to pellet the RBCs. The supernatant was carefully aspirated without disturbing the pellet and 40 µl of cold PBS was added to each sample and after mixing, the contents transferred to a microtiter plate. Samples were either processed immediately or stored at −20°C pending analysis. Immediately prior to use, LDH buffer (100 mM Tris-HCL, 10 mM sodium lactate, 0.25% Triton X-100, and pH 7.5) was supplemented with nitroblue tetrazolium (NBT) 0.5 mg/ml, APAD (0.05 mg/ml) and phenazine ethosulfate (PES) (0.02 mg/ml). A 100 µl volume of the complete LDH buffer was added to each well, the plate placed on a rocking platform and the reaction allowed to proceed for 40 min in the dark and absorbance read at 650 nm using a multi-scan plate reader.

### Data analysis

Statistical analysis of the data was performed using SPSS 8.0 software (SPSS Inc., Chicago, Illinois). The prevalence of anemia was compared according to WHO age/gender based classification and category of infection: not infected (N), schistosome only infected (S), malaria only infected (M) and schistosome and malaria co-infected (S+M) using Chi-square tests. The mean hemoglobin concentration was compared using analysis of variance (ANOVA) and either a t-test (with Bonferroni correction) or the Mann-Whitney test. Statistical significance was designated as P<0.05.

## Results

### Baseline diagnosis, schistosome burden and criteria for subject selection

**Figure 2:**
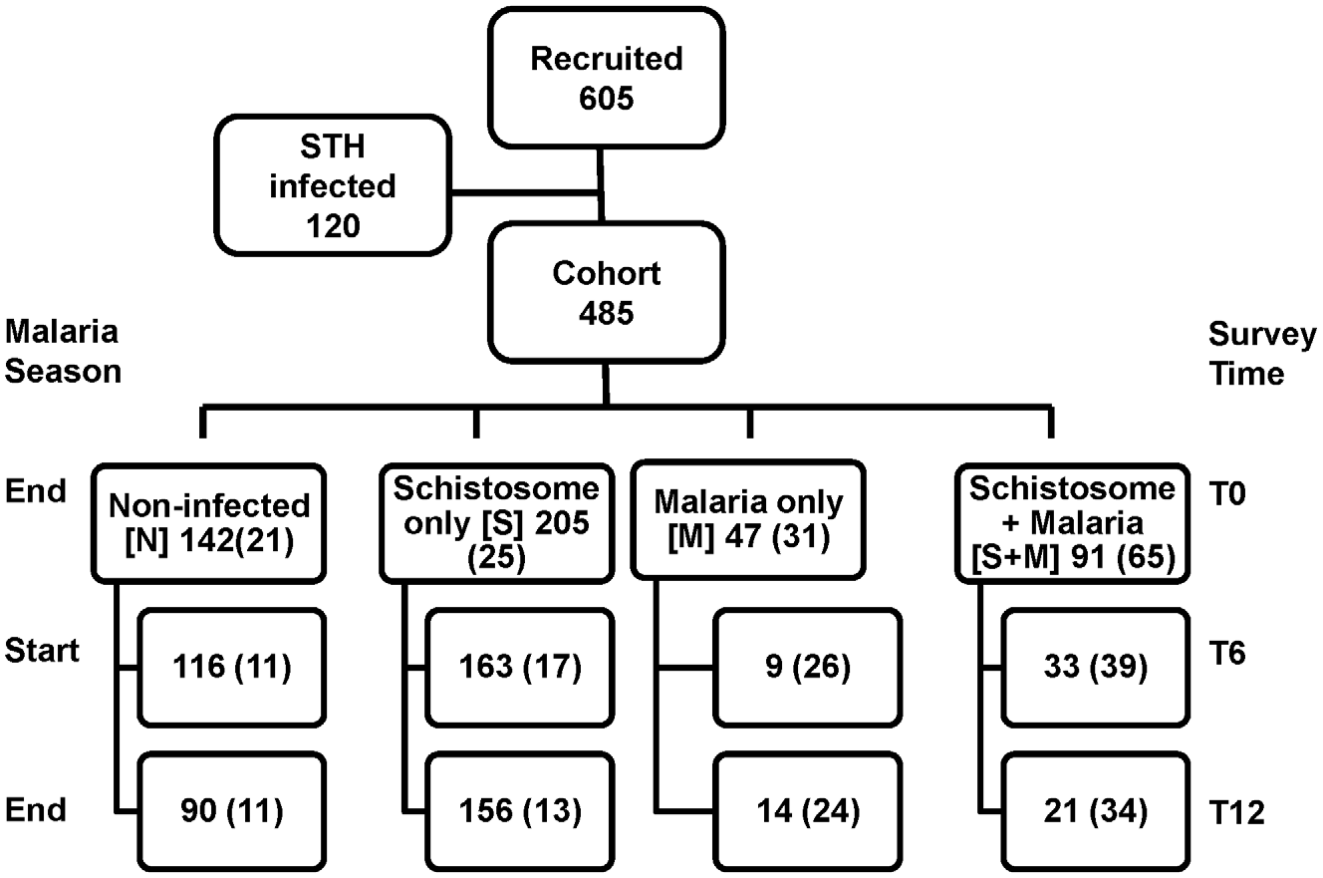
Numbers of Recruited Participants and Cohort. Recruitment, loss to follow up and the follow up of cohort after grouping by diagnosis at baseline survey (T0) and the malaria season status are shown. The numbers of subjects grouped into various diagnostic groups were 485 at T0, 321 at T6 and 281 at T12. At T6 and T12 the numbers of children that tested positive for malaria only were 9 and 14, respectively and those tested positive for malaria and schistosomes were 33 and 21, respectively. A subset of subjects from each of the 4 categories based on the baseline diagnosis was maintained at subsequent follow-up time points T6 and T12. These subjects (numbers in parentheses) were chosen based on the availability of adequate serum volumes for follow-up laboratory studies. Only a representative fraction from the uninfected (N) and schistosome only (S) infected children were included in analyses due to limited reagent quantities, especially recombinant antigens used in ELISA.

We recruited 605 children (299 girls and 306 boys) at the baseline survey. However, a total of 120 children of the 605 were excluded due to presence of other helminth infections or could not be accurately assigned to any diagnostic group due to missing diagnostic data. Figure 2 shows the numbers of children that had adequate blood samples for analyses at T0, T6 and T12. A total of 485 children provided sufficient samples at baseline (T0) for malaria and schistosomiasis diagnosis and could be classified into 4 categories; not infected (N), schistosome-only infected (S), malaria-only infected (M), and schistosome and malaria co-infected (S+M) and constituted the enrolled cohort (Figure 2). At T6 and T12 the numbers of children examined were 321 and 281, respectively. However, only about a third of these had complete diagnostic information and even fewer provided adequate serum samples to allow antibody and GIA analyses as discussed in this study. The samples at T0, T6 and T12 that were available for analyses are shown in parentheses (82 subjects shown at T12 representing the cohort). The distribution of *S. haematobium* and *S. mansoni* infections among the study subjects at T0 is shown in Table 1. *S. haematobium* infection accounted for 51.3% (249/485/), *S.mansoni* 8.5% (41/485), whereas, a combination of both infected 13.2% (64/485) of the children and 27% (131/485) were schistosome free (Table 1). There was also a significant difference in *S. haematobium* egg counts per 10 ml of urine between malaria infected (median  = 109) and malaria free (median  = 68) children (p = 0.001 Mann-Whitney Test). On the other hand there was no significant difference in *S. mansoni* egg counts between *S. mansoni* and malaria infected (median  = 112) and malaria free (median  = 106) children (p = 0.4).

**Table 1:** Distribution of *S. haematobium* and *S. mansoni* infection among the study subjects at T0.

		*S. mansoni* infection status at T0		Total
		(−)	(+)	
*S. haematobium* infection status at T0	(−)	131	41	172
	(+)	249	64	313
	Total	380	105	485

Wherever possible, all available data on children were utilized during analysis, however, in some situations subsets of data were used based on availability of adequate samples. In addition to loss to follow-up, not all samples had adequate blood samples for all diagnostic tests for all children at the subsequent follow-up time points. Baseline diagnostic groups were maintained across follow-up time points for comparison with baseline although the infection status (malaria and schistosomiasis) may have changed at subsequent follow-up time points. Figure 2 shows that the final numbers of children with the complete data at last follow-up time T12 was 82 (45 boys and 37 girls) corresponding to various diagnostic groups, N (11), S (13), M (24) and S+M (34). The mean age of children in the cohort was approximately 10 years as was that of the study population.

### The effect of schistosome infection on malaria prevalence at T0, T6 and T12

**Figure 3:**
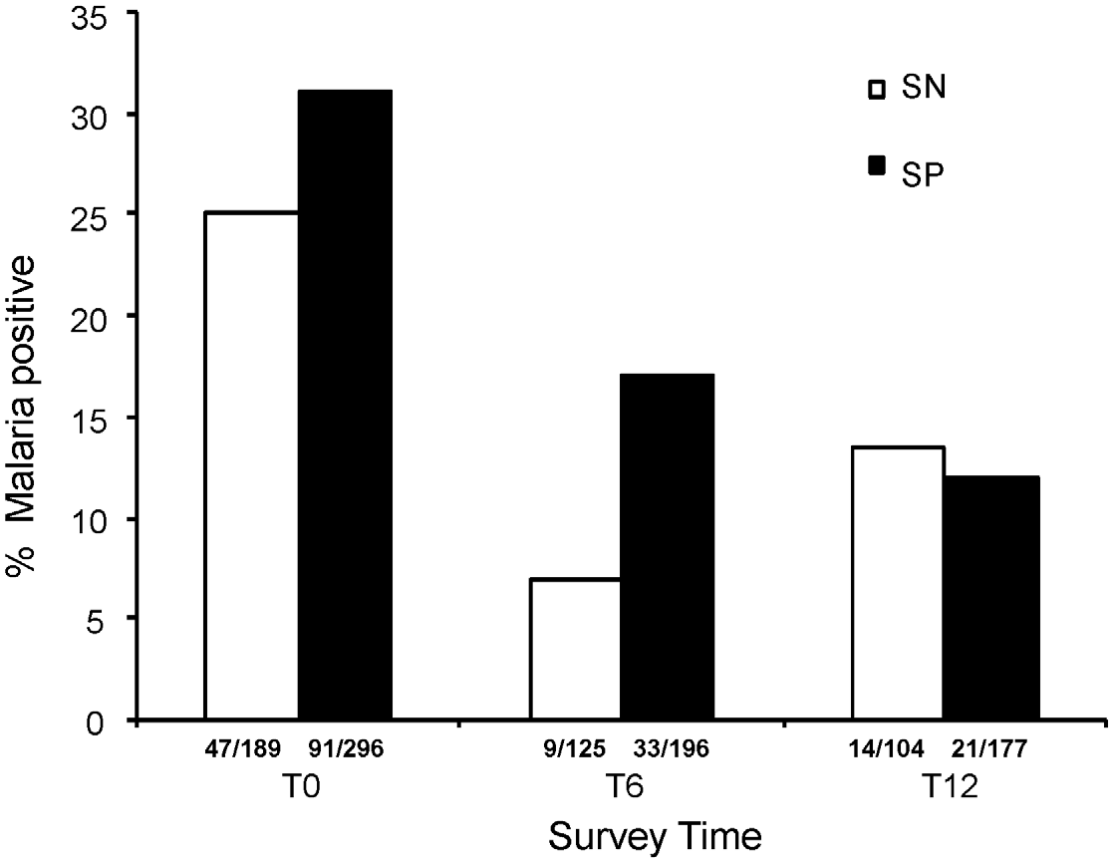
Effect of schistosome infection on malaria prevalence. Total number of children testing positive for malaria at T0, T6 and T12 among schistosome positive (SP) and negative (SN) are shown on x-axis. The bars show the percentage values for each survey time point. A higher proportion of schistosome positive children (black bars) had malaria parasites at T0 (p = 0.064) and T6 (p = 0.053) compared to the schistosome negative children (white bars). At T12 the malaria prevalence was not different for the two groups.

Schistosome-infected subjects were treated with praziquantel at each time point, although only a few subjects were re-infected or newly infected. *P. falciparum* malaria infection prevalence was found to be higher among *S. haematobium* infected subjects compared to their schistosome-free counterparts at T0 (31% versus 25%) (p = 0.064) and T6 (17% versus 7%) (p = 0.053) (Figure 3). However, this difference diminished at T12 as the malaria prevalence levels observed were 12% for schistosome-infected children and 13.5% for schistosome-free children. On the other hand, *S. mansoni* infection was not significantly associated with malaria prevalence (p = 0.87).

### Prevalence and geometric mean parasite densities of asexual and sexual stages of *P. falciparum*

**Figure 4:**
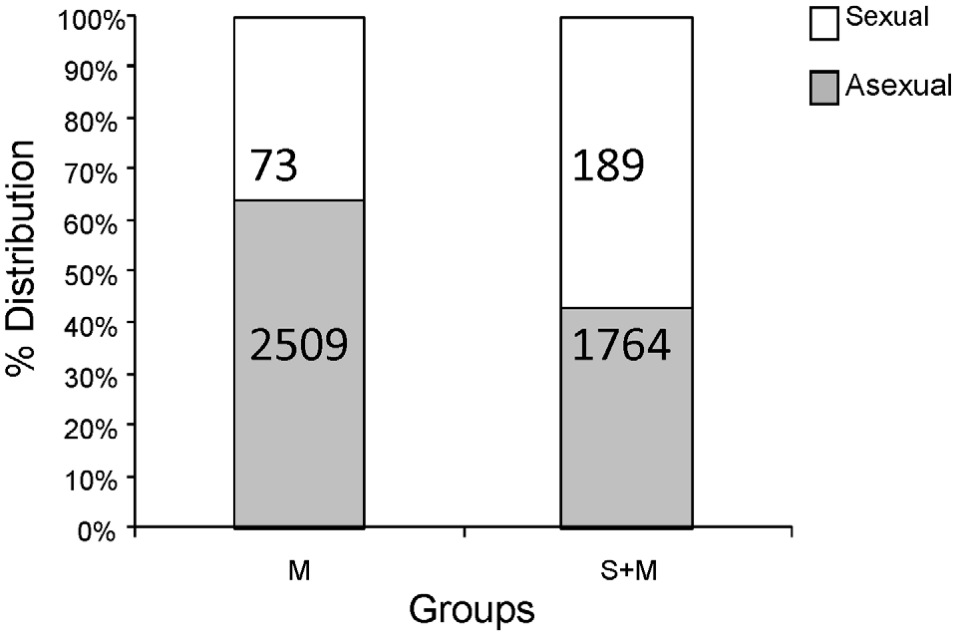
Percentage distributions and geometric mean parasite densities of sexual (gametocyte) and asexual (ring) parasites in malaria-infected children. Numbers inside bars represent geometric mean parasite density values for sexual (white bars) and asexual (black bars) in the M (n=14) and S+M (n=28) groups for a subset of subjects for which these data were available. Co-infected children had a higher proportion of gametocyte infection (p=0.06) and higher mean gametocyte density in blood (p=0.043) compared to malaria only infected children (M).

A subset of available samples was analyzed for further estimation of prevalence and density of asexual and sexual forms in the M and S+M groups. At T0 only 36% of the malaria-only infected (M) children had gametocytes by microscopy whereas 57% of the co-infected (S+M) (p = 0.06) children had gametocytes (Figure 4). It thus appears that schistosome and malaria co-infection may be associated with higher prevalence of gametocytes in peripheral blood of subjects in this population. Not only did we observe a tendency for a higher proportion of children with gametocytes during co-infection (p = 0.06, approaching significance), but we also noted significantly higher (p = 0.05, Mann-Whitney test) gametocyte geometric mean parasite density during co-infection (189 gametocytes/µl of blood in S+M group compared to 73 gametocytes/µl in M group). Conversely, the mean density of asexual parasites (ring forms) in co-infected children was lower than that of malaria only infected children (1764 rings/µl in S+M group (Figure 4) compared to 2509 rings/µl in the M group. However, this difference was not statistically significant (p = 0.41).

### Hemoglobin levels and proportion of children with anemia at different time points

**Figure 5:**
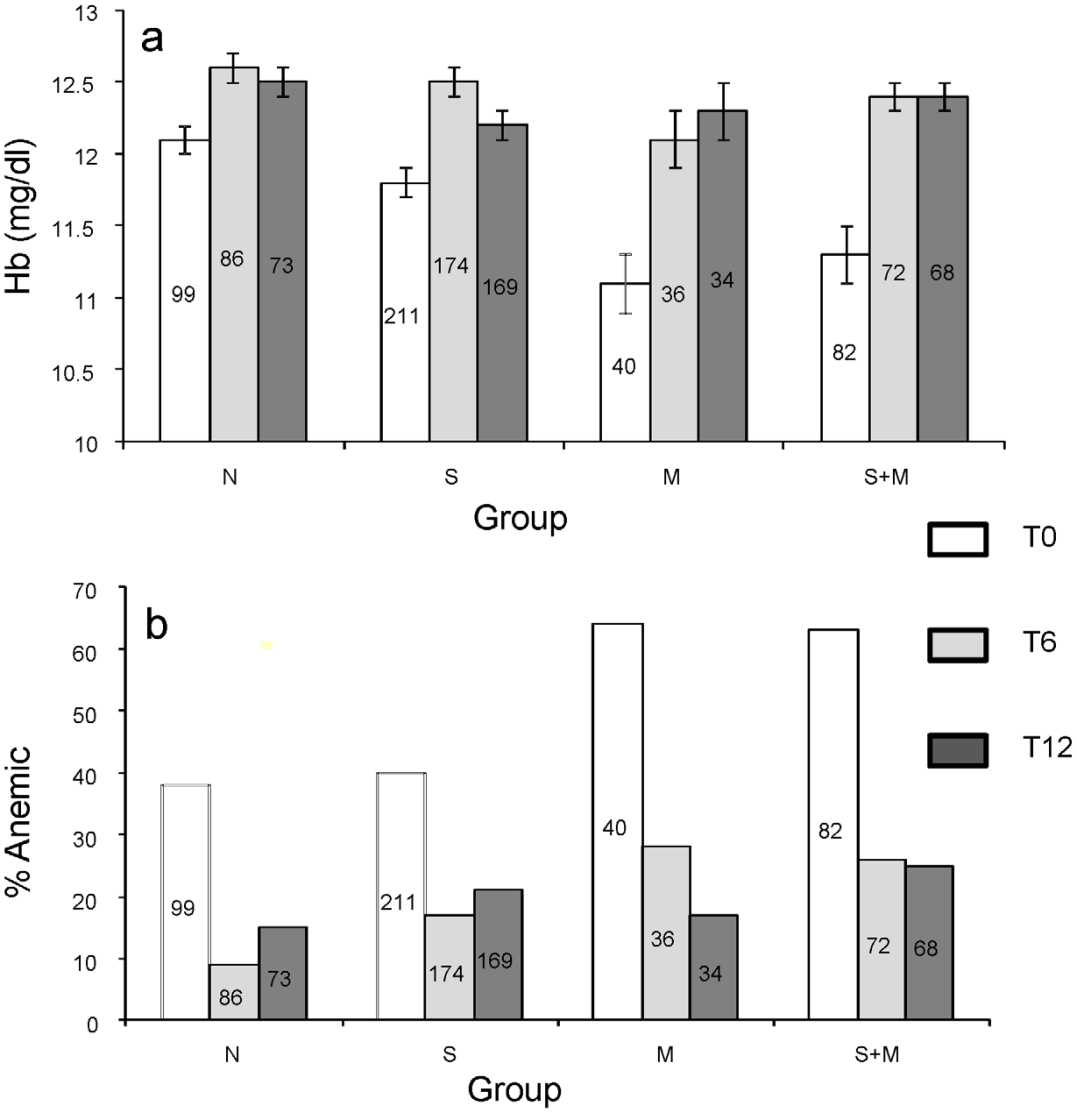
Mean hemoglobin levels and prevalence of anemic children for each diagnostic group at different sampling times. At T0, differences in the hemoglobin levels (Figure 5a) in groups M and S+M were statistically significant (p<0.001 by t-test) as compared to group N. A comparison between M and S+M and groups N and S revealed non-significant p values, 0.743 and 0.183, respectively. Figure 5b shows results of proportion of anemic children in various groups, p values at T0 for differences between groups N and M, groups N and S+M and groups N and S were 0.01, 0.001, and 0.273, respectively. The numbers in each bar represent ‘N’ for each group and the error bars represent SEM.

At recruitment (T0) the baseline mean Hb levels of the four diagnostic groups showed differences (F = 5.048, p<0.0001). Schistosome infection by itself did not result in a significant drop in mean Hb levels and malaria infection exerted the greatest impact on Hb levels (Figures 5a). The differences in Hb levels were significant between N and M groups (p = 0.0001), N and M+S groups (p = 0.0001), but not between N and S (p = 0.183). All infected children at T0 received appropriate treatment and there was no significant difference detected by ANOVA in mean Hb levels of the different diagnostic groups at T6 (F = 1.520, p = 0.189) nor at T12 (F = 0.143, p = 0.982).

According to WHO age and gender-based anemia classification, at T0 38% of the children in group N, 40% in group S, 64% in group M and 63% in group S+M were categorized as anemic. The proportion of anemic children at T0 in the groups M and S+M as compared to group N were significantly higher with p = 0.0001 and p = 0.01, respectively. At T6 and T12 there was no statistically significant differences in anemia levels across diagnostic groups (Figure 5b).

### Total IgG reactivity to crude *P. falciparum* antigen at different time points and to MSP-1 and AMA-1 at T0

**Figure 6:**
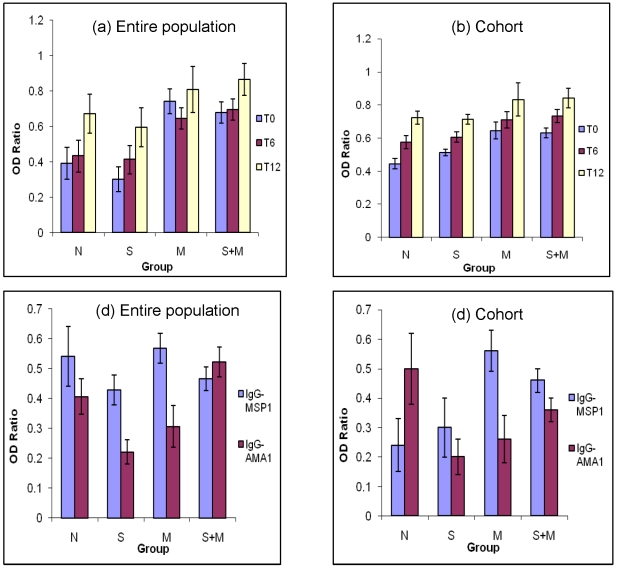
Comparisons of mean OD ratio of IgG to crude and specific *P. falciparum* antigens at different time points. The antibody levels were determined by ELISA from serum samples from children in various diagnostic groups. Generally antibody activity increased with time from T0 to T12 with higher activity in children who had malaria at baseline. Total number of serum samples for data on entire population in Figure 6a were T0 (n = 446), T6 (n = 342), and T12 (n = 310). Figure 6b shows data on 82 children in the cohort at all three time points (T0, T6, T12). Figure 6c shows antibody reactivity results of population sera to MSP-1 (n = 193) and AMA-1 (n = 198) at T0. Figure 6d shows results for cohort children to MSP-1 (n = 79) and AMA-1 (n = 81) at survey T0. Comparison of OD ratios between groups M and S+M or groups N and M for AMA-1 revealed p values of 0.006 and 0.001, respectively by Mann-Whitney test. Error bars represent SEM.

We assessed the mean levels of antibody reactivity to crude malaria antigen extract for all available plasma samples (entire population) as well as for the cohort (n = 82) for each diagnostic group at the three different time points. Plasma IgG reactivity to crude *P. falciparum* antigen extract generally increased with time over the one-year study period in all groups. The antibody levels were significantly different among groups at T0 (F = 8.169, p<0.001) andT6 (F = 2.633, p = 0.05) but subsequently exhibited no difference in antibody activity to malaria antigens among the groups at T12 (F = 1.162, p = 0.280) (Figures 6a and 6b). At T0, the p values (t-test) for various comparison groups (indicated in parenthesis) were 0.064 (N and S), 0.001 (N and M), <0.001 (N and S+M) and 0.946 (M and S+M). Likewise, p values at T6 were 0.579 (N and S), 0.05 (N and M), 0.018 (N and S+M), and 0.861 (M and S+M). No significant differences were found at T12 among various comparison groups. In all cases OD ratios were used instead of absolute OD values in order to normalize results for plate to plate variation and negative and positive controls were concurrently run on each plate to ensure plate to plate assay consistency.

**Figure 7:**
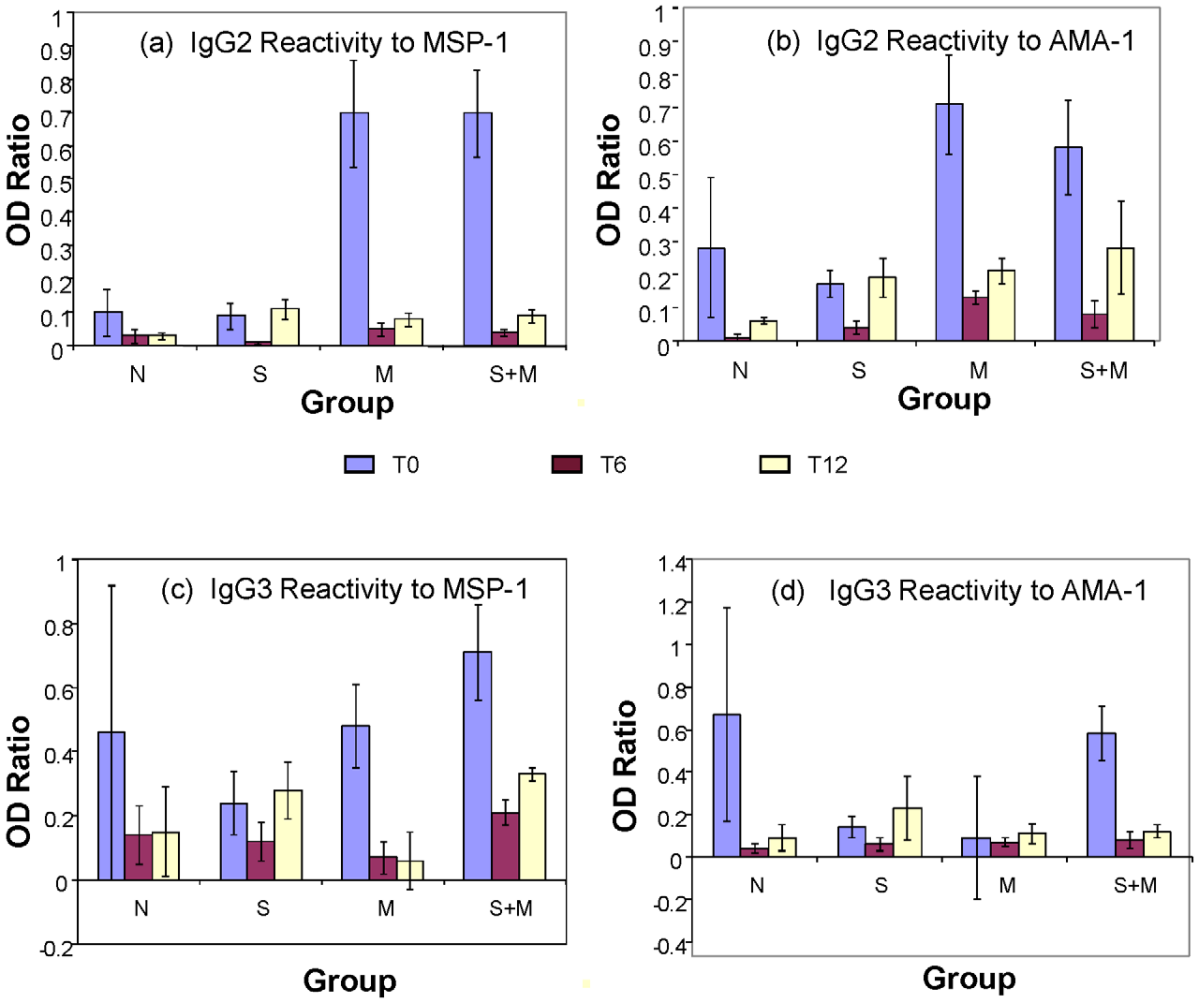
Comparisons of IgG2 and IgG3 isotype-specific antibody activity in serum from subjects in different groups. Cohort sera samples from three time points (T0, T6 and T12) were analyzed by ELISA to detect IgG2 and IgG3 isotype-specific antibody activity to MSP-1 (Figures 7a and 7c, respectively) and AMA-1 (Figures 7b and 7d, respectively). Error bars represent SEM.

In order to determine whether there were any differences in plasma levels of antibody reactivity to specific antigens (MSP-1 and AMA-1) at T0, we repeated the ELISA to determine relative antibody levels in different groups with plates coated with the purified recombinant MSP-1 and AMA-1 antigens (Figure 6c entire population and Figure 6d for cohort). These assays did not reveal any specific patterns among the 4 groups except that anti-MSP1 antibodies in group M were highest as compared to malaria uninfected groups and that there were no appreciable differences between M and S+M groups (Figures 6c and 6d). The same assay could not be performed for T6 and T12 samples due to limiting amounts of AMA-1 and MSP-1 antigens available. We also analyzed serum samples for antigen specific antibody isotypes in the sera samples from children in the cohort and found that IgG2 antibody reactivity to both specific malaria antigens (MSP-1 and AMA-1) in the malaria-infected groups (M and S+M) was markedly increased compared to the reactivity of malaria-free control groups (N and S) at T0 ( Figures 7a and 7b). Globally there was a significant difference in antibody activity among the diagnostic groups p < 0.0001 and individually between either of the malaria infected groups and the uninfected group, N using the Kruskal-Wallis test at T0 but not at T6 and T12. At T0, IgG3 isotype antibody reactivity to MSP-1 also differed among diagnostic groups by ANOVA Subsequent time point reactivity differences were not significant across groups (Figures 7c and 7d). We also investigated the levels of cytokines IL-10 and IFN-γ in the serum samples from the various diagnostic groups and did not find any differences in patterns (data not shown).

### In vitro growth inhibition assays

**Figure 8:**
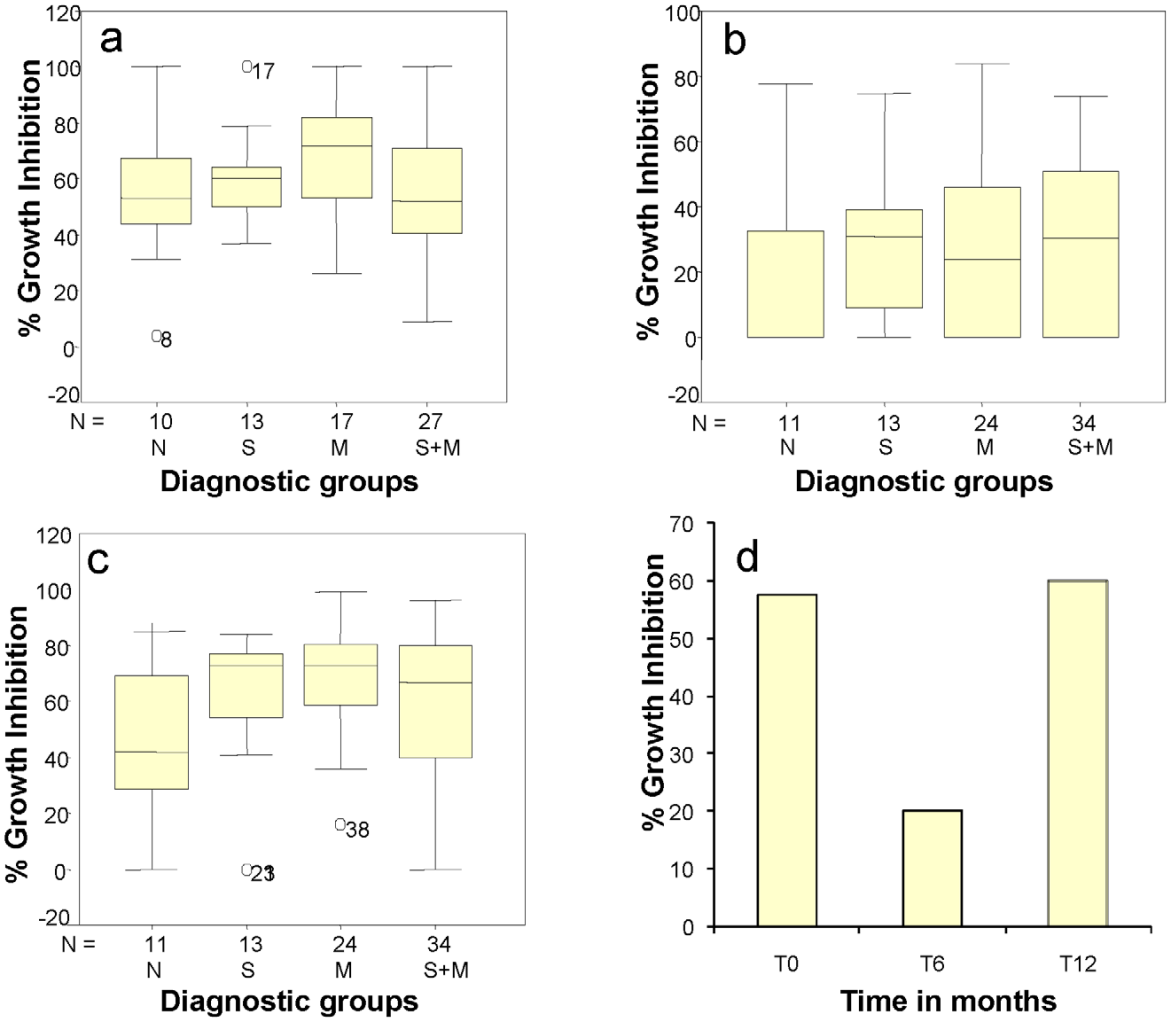
Mean growth inhibition activity of different groups at different malaria seasons and survey times. Ability of subjects' plasma samples to inhibit *P. falciparum* growth from each group at T0 (Fig. 8a), T6 (Fig. 8b) and T12 (Fig. 8c) are shown. The box plots display *in vitro* percentage growth inhibition activities of *P. falciparum* by participants' plasma samples (at 10% concentration) after 48 h growth of synchronized W2 culture. The middle horizontal line in each box indicates the median percentage growth inhibition for each diagnostic group, and the box indicates the 25^th^ and 75^th^ percentiles. The whisker caps extending from each box indicate the minimum and maximum values. Individual marked points represent a few outlier values. Figure 8d represents mean growth inhibition data for the entire cohort at three transmission seasons (T0, T6, T12).

Generally, the total immunoglobulin–specific activity of plasma from individuals may not be indicative of the level of protection against malaria, though the level of IgG3 and in some cases IgG1 has been significantly associated with relative protection from clinical *P. falciparum* malaria attacks [27]. In order to compare the functional activity of antibodies from individuals belonging to different diagnostic groups we carried out GIAs with the individual plasma samples and calculated the median growth inhibition potential for each diagnostic group. At baseline (T0) the highest median percentage inhibition was for the Group M (70%), though it was not significantly higher than the other groups that were at 50–60% growth inhibition (Figure 8a). At T6, the percentage growth inhibiting capacity declined precipitously in all groups especially in Group N which went down to 0% inhibition whereas all other groups exhibited a 40–50% decline in percent *in vitro* growth inhibition (Figure 8b). At T12, 6 months later the percent *in vitro P. falciparum* growth inhibiting potential was restored to a level, a slightly higher than it was at To (Figure 8c), most likely as a result of re-exposure to malaria during ensuing transmission season. Figure 8d shows a direct comparison of overall mean growth inhibition capacity of all subjects' serum samples at T0, T6 and T12 for the full cohort demonstrating a rapid loss of growth inhibition capacity of sera at the end of malaria transmission season. The mean values at T6 were significantly different (p = 0.04) from those at T0 and T12.

## Discussion

Our results suggest increased malaria susceptibility during helminth infection and are in agreement with many studies which found that helminths increased susceptibility to malaria [5], [7]–[11]. However, other studies found null effect [12]–[14], and still others reported lower rates of malaria infection (protection) [15]–[18]. A subset of available samples was analyzed to further characterize differences between asexual and sexual forms in the M and S+M groups. At T0 only 36% of the malaria-only infected (M) children had gametocytes whereas 57% of the co-infected (S+M) children had gametocytes suggesting that schistosome and malaria co-infection is associated with a trend towards higher prevalence of gametocytes in peripheral blood of subjects in this population (p = 0.06). Our results are in agreement with those reported by Nacher and colleagues who found that helminth infections were associated with patent gametocyte carriage in Thailand [42] while Drakeley and colleagues observed a significant positive association between packed cell volume, reticulocyte count and lymphocyte count with peripheral blood gametocyte density in malaria outpatients in The Gambia [43]. Further analysis of the same subset of samples revealed not only elevated proportion of children with gametocytes during co-infection, but also significantly higher gametocyte geometric mean parasite densities (189gametocytes/µl) compared to 73 gametocytes/µl of the malaria-only infected group (p = 0.05). It is worth noting that the observed differences in gametocyte prevalence and density were based on microscopic analysis. The precise reasons for increased gametocyte carriage in peripheral blood during helminth co-infection remain largely unknown. The implications of the higher peripheral gametocytemia during co-infection on the potential for malaria transmission require further study using more sensitive RT-PCR gametocyte detection methods and mosquito membrane feeding assays which can establish actual infectivity of gametocytes in the blood and transmission differences.

A pre-existing helminth infection may predispose children to development of gametocytemia through events leading to anemia and tissue hypoxia, which may stimulate *P. falciparum* gametocytogenesis as a survival strategy [42], [44]. The physiology of gametocytogenesis however, is still incompletely understood. In another study, Nacher and colleagues reported that hemoglobin levels were lower in gametocyte carriers, peak gametocytemia and carriage durations were negatively correlated with hemoglobin concentration, median asexual parasitemia and severe malaria cases were associated with higher gametocytemia compared to mild malaria following adjustment for confounding variables [45].Whatever the possible causes, helminth co-infections tend to be positively correlated with higher gametocytemia and also a tendency for higher proportion of co-infected children carrying gametocytes.

At baseline mean Hb levels among the diagnostic groups were statistically significantly different but were no longer significant at T6 and T12 following treatment of schistosome infected children with praziquantel. At T6 the similarly high hemoglobin level may be attributed to the fact that the children were generally malaria free in the intervening six months in which helminth infections had also been cleared from the children by chemotherapy. Malaria appeared to be the primary cause of anemia and showed no synergistic or antagonistic interaction with schistosomiasis during co-infection. Generally the proportion of anemic children was significantly higher at baseline compared to the proportion anemic at subsequent follow-up times.

In the present study we also investigated the qualitative and quantitative differences in various immune parameters in different groups of children. Generally, the total IgG-specific activity and levels showed a gradual increase from T0 to T12 reflecting a likely increase in malaria specific antibody levels expected in an endemic area. However, at each time point the malaria-infected group demonstrated significantly higher specific activity compared to the malaria-free groups. At T0 in the entire study population IgG levels to MSP-1 were also higher in malaria infected subjects compared to their malaria free counterparts (p = 0.0001).

Recent studies have reported cross-reactivity of IgG3 isotype antibodies to *P. falciparum* schizonts and *S. mansoni* egg antigen(SEA) [46]. In this study we also attempted to seek a correlation between malarial antigen specific IgG3 antibodies and various infection groups. While our preliminary results revealed a trend for elevated IgG3 isotype antibodies to MSP-1 and AMA-1 in S+M group, we do not know if these IgG 3 also cross-react with schistosome antigens. A systematic analysis involving affinity purified IgG3 antibodies may further clarify any such possibility.

There was no statistically significant difference among the diagnostic groups in terms of growth inhibition capacity at T0 (p = 0.406), T6 (p = 0.602) and T12 (p = 0.271) by the Kruskal- Wallis test. An interesting trend was revealed by the evaluation of the subjects' plasma in GIA. At T0 (the end of one malaria season) the mean growth inhibition activity of plasma samples from all children included in the analysis was 58% but dropped to 20% at T6 (at the beginning of the next malaria season) and rose back to more than 60% at the end of the next malaria season at T12 (Fig. 8d). It is suggested from the box plots that greater growth inhibition capacity occurred in the malaria-only infected group and following an intense malaria transmission season, though this did not reach statistical significance with our current sample size. This phenomenon deserves to be explored with a larger study population. The growth inhibition activity of plasma from co-infected children tended to be lower than that of malaria-only infected children although antibody levels to malaria antigens were similar between the two groups at T0. Future investigations are warranted to determine whether the antimalarial immunoglobulins in the helminth infected malaria subjects are qualitatively different from those in malaria-only subjects. This fluctuation of growth inhibition potential seems to suggest that malaria immunity depends on recent prior exposure to malaria infection. At T6, at the start of the next malaria season, there was a dramatically lower level of malaria parasite growth inhibition capacity as measured by in vitro GIA across all groups. This seems to suggest that the GIA capacity requires consistent exposure to malaria parasites in order to remain effective. This drop in growth inhibition potential occurred despite the rise in IgG specific activity at T6, suggesting once more that it is not a simple function of higher antibody activity or levels only. At T12, at the end of another malaria season, the growth inhibition activity rose across all the diagnostic groups once again suggesting a temporal relationship between immunity to malaria and malaria parasite exposure.

In conclusion, in the present study it was evident that schistosome co-infection with malaria positively corresponded with higher incidence and density of gametocytes which likely leads to more intense malaria transmission. In children with higher schistosome egg burdens of *S. haematobium* infection was associated with higher prevalence of malaria. There was neither a significant difference in egg burdens nor malaria prevalence in *S. mansoni* infected children. Malaria-infected subjects in this population had lower hemoglobin levels than their malaria-free counterparts and schistosome infection did not exacerbate anemia. Specific IgG activity and levels showed a gradual increase over time from T0 to T12 reflecting increase in malaria specific antibody levels as expected in an endemic area. Plasma from malaria-infected subjects showed higher malaria antibody activity in response to malaria antigens. Growth inhibition activity of plasma was higher for malaria-only infected children than that from co-infected children though antibody levels were comparable for the two groups. Mean GIA of plasma samples from all children included in the analysis was higher at the end than at the beginning of malaria seasons. Such fluctuations may need to be taken into account when evaluating vaccine efficacy in areas of seasonal malaria transmission. The most notable impact of schistosome co-infection was revealed by the prevalence of sexual and asexual stage parasites which may have implications on malaria disease severity and transmission dynamics.
